# Carcinoma in situ within the bladder trigone with an isolated metastasis to the prostate without involvement of prostatic urethra: a unique case report

**DOI:** 10.1186/s12894-024-01516-6

**Published:** 2024-06-15

**Authors:** Omran Batha, Ahmed Aldolly, Yousef Alsaffaf, Suaad Hamsho, Mohammad Atia, Fayez Salmeh, Louei Alia

**Affiliations:** 1 Damascus University, Al-Assad University Hospital, Damascus, Syria; 2 Faculty of Medicine, Hama University, Assi Square, Hama, Syria; 3 Rheumatology Department, Faculty of medicine, Damascus University, Damascus, Syria; 4 Al-Sham private university, Damascus, Syria

**Keywords:** Carcinoma in Situ, Bladder, Prostate, Prostatic Urethra, Metastasis, Case Report

## Abstract

**Background:**

Carcinoma in situ of the bladder is a high-grade cancer that originates in the superficial layer of the bladder. It has the potential to invade nearby organs, and it can spread through blood and lymphatic circulation to distant parts of the body.

**Case presentation:**

A 58-year-old non-smoker male presented with gross and microscopic hematuria. His family history included his father’s recent bladder cancer. Initial investigations showed hematuria, inflammation, negative urine culture, digital rectal examination revealed an enlarged right lobe of the prostate, and an elevated Prostate-Specific Antigen level. Histopathological examination of samples taken from the bladder mucosa and the prostate confirmed urothelial carcinoma in situ in the bladder and prostate. Further evaluation revealed no other metastasis. The tumor was classified as T4aN0M0. The patient underwent radical cystoprostatectomy and histopathological examination showed that the tumor invading the muscularis propria of the bladder as well as the prostatic glands, but no malignancy was found in prostatic urethra and other areas. The patient was discharged three weeks post-operation and completed on adjuvant chemotherapy consisting of Gemcitabine, and Cisplatin to prevent of relapse. The patient is currently in a good healthy.

**Conclusion:**

The occurrence of bladder cancer metastasizing to the prostate without involving the prostatic urethra is uncommon and requires precise diagnostic techniques for accurate tumor classification. Early management is advised to enhance the prognosis for the patient.

## Background

Carcinoma in situ (CIS) of the bladder is a type of cancer that originates in the superficial layer of the bladder and can appear singly or multiple. It is a high-grade cancer that can invade nearby organs such as the kidneys, pelvis, prostate, vagina and can metastasize through blood and lymph circulation to distant parts of the body [1–3]. Individuals across all age groups can be affected, yet it’s more prevalent in those over the age of 40. Aging is one of the significant risk factors that raise the likelihood of acquiring the condition. Additional risks include contact with certain chemicals like arsenic and those found in leather dyes. Furthermore, smokers are at a substantially higher risk, with about 75% experiencing more than a fivefold increase in the likelihood of developing the condition compared to non-smokers [1, 3]. The prognosis of this type of cancer varies based on multiple variables such as tumor size, tumor type, and the patient’s responsiveness to treatments like chemotherapy, radiation, and surgery. However, the five-year survival rate is usually higher than 50% [1]. In this paper, we discuss a case of a CIS of the bladder with prostate metastases in an unusual way without any signs of malignancy in prostatic urethra.

## Case presentation

**Fig. 1:**
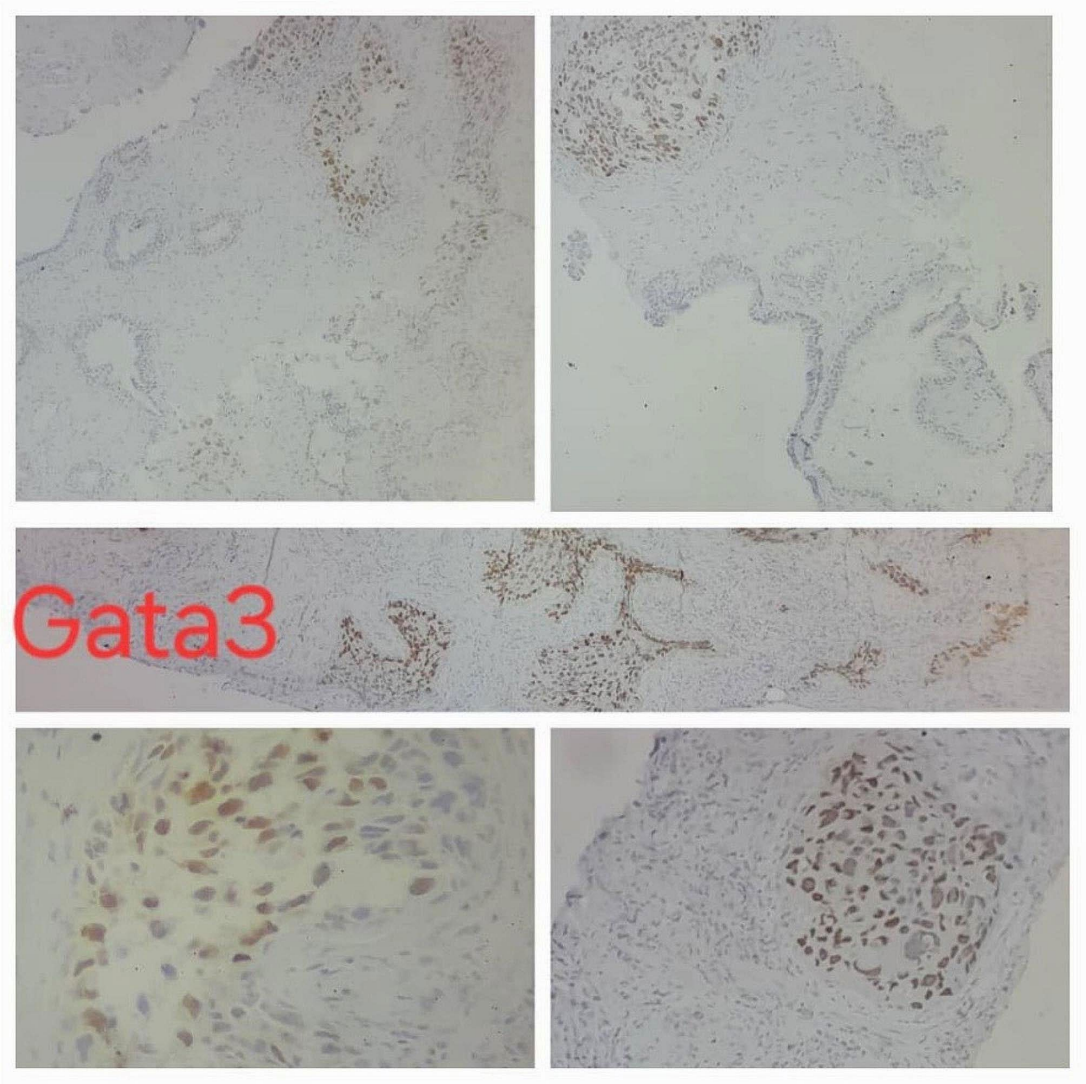
Prostate biopsy revealed high-grade carcinoma in prostatic ducts, immunohistochemical staining resulted in negative NKX3.1 and positive Gata3 markers, indicative of a primary urothelial origin of the cancer

**Fig. 2:**
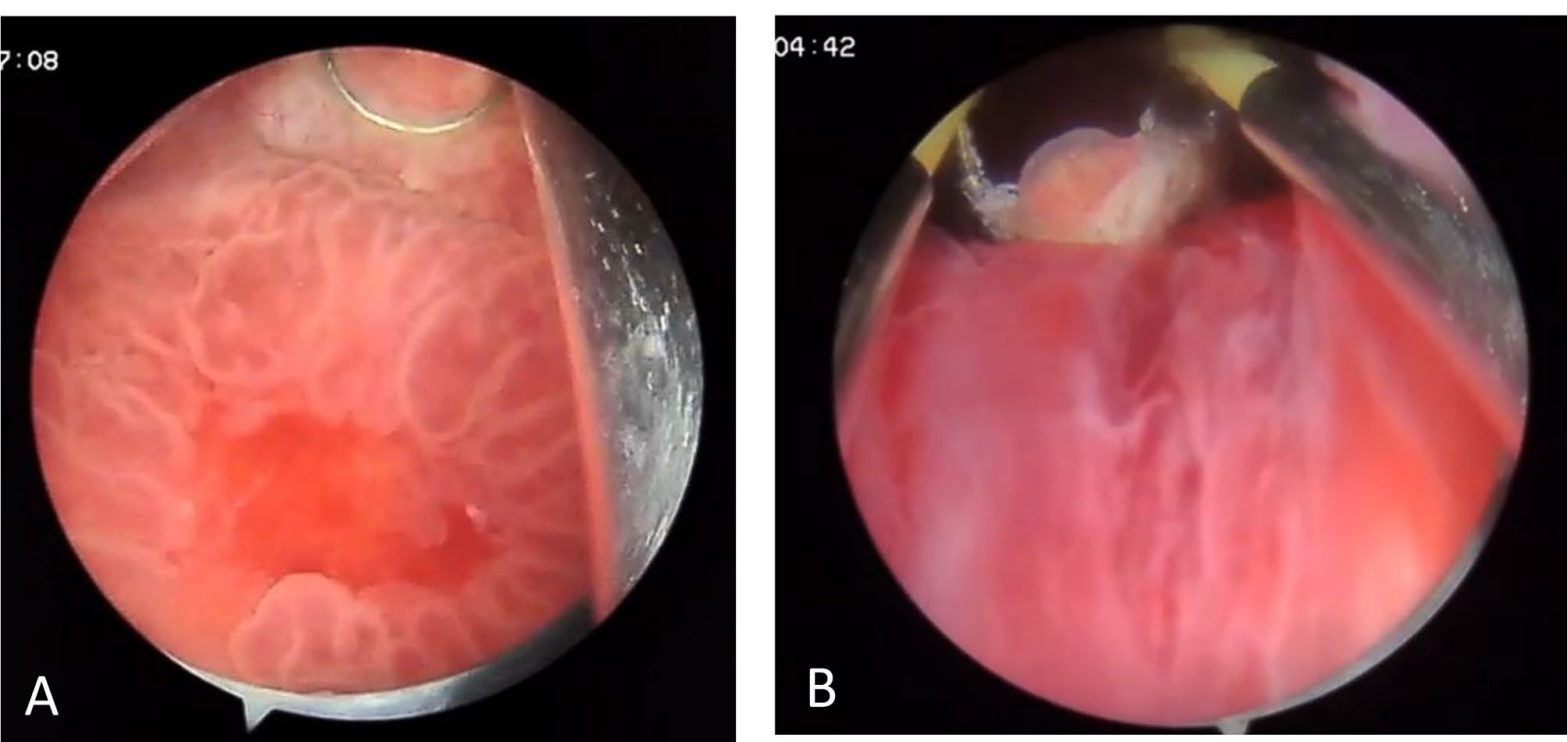
**(A)** The cystoscopy revealed edematous bladder mucosa with red areas and minor bleeding from the prostate. No visual signs of malignancy were observed. **(B)** The cystoscopy revealed normal appearance of the prostatic urethra during visual examination

**Fig. 3:**
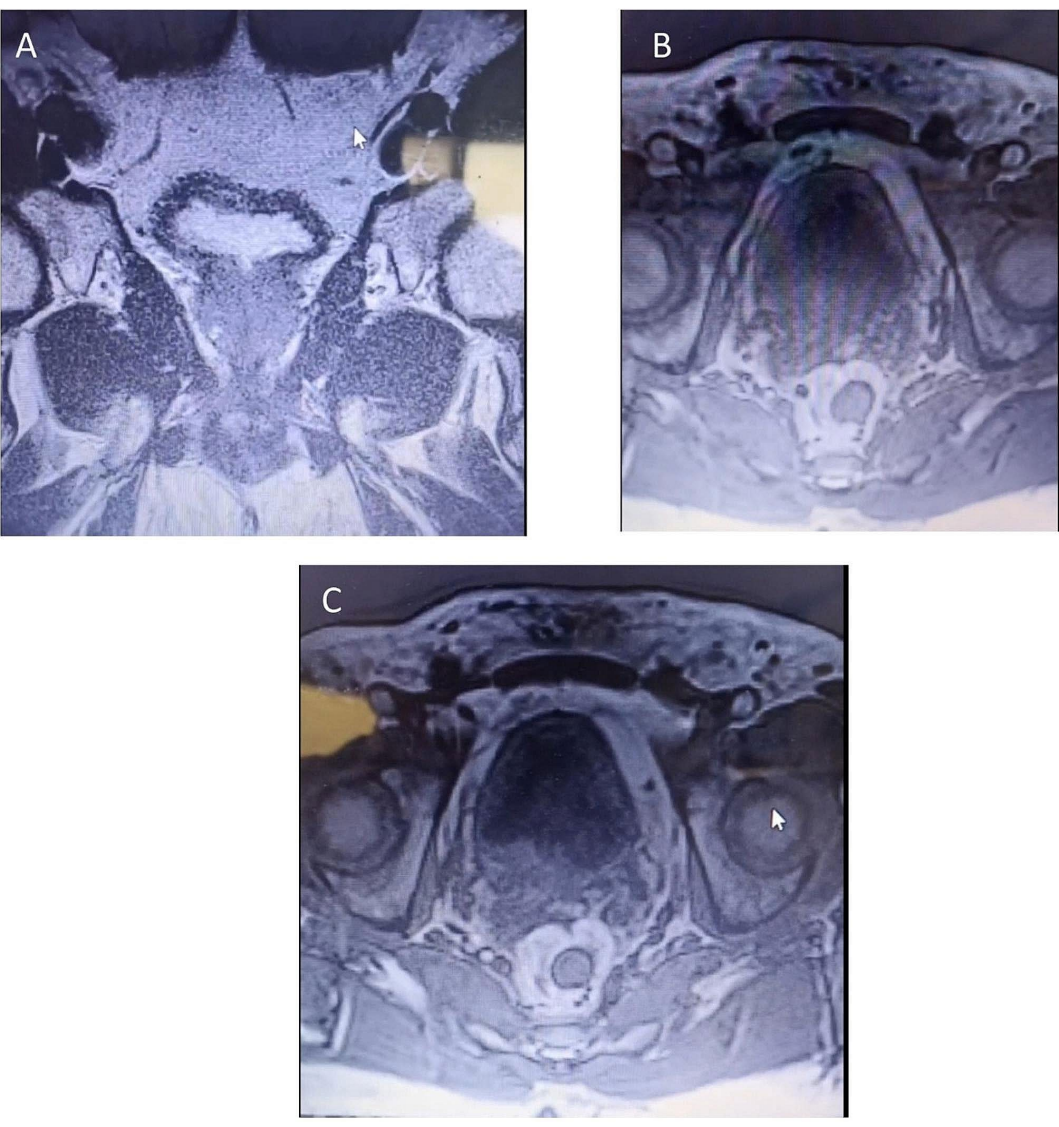
MRI revealed **(A)** a lesion exhibiting high signal intensity on T1-weighted images within the right peripheral zone of the prostate. This finding suggests the presence of a possible tumor or hemorrhage, which could be a post-biopsy complication. (**B**, **C**) A slight thickening of the bladder wall mucosa was observed post-injection, suggesting potential inflammatory or neoplastic alterations

**Fig. 4:**
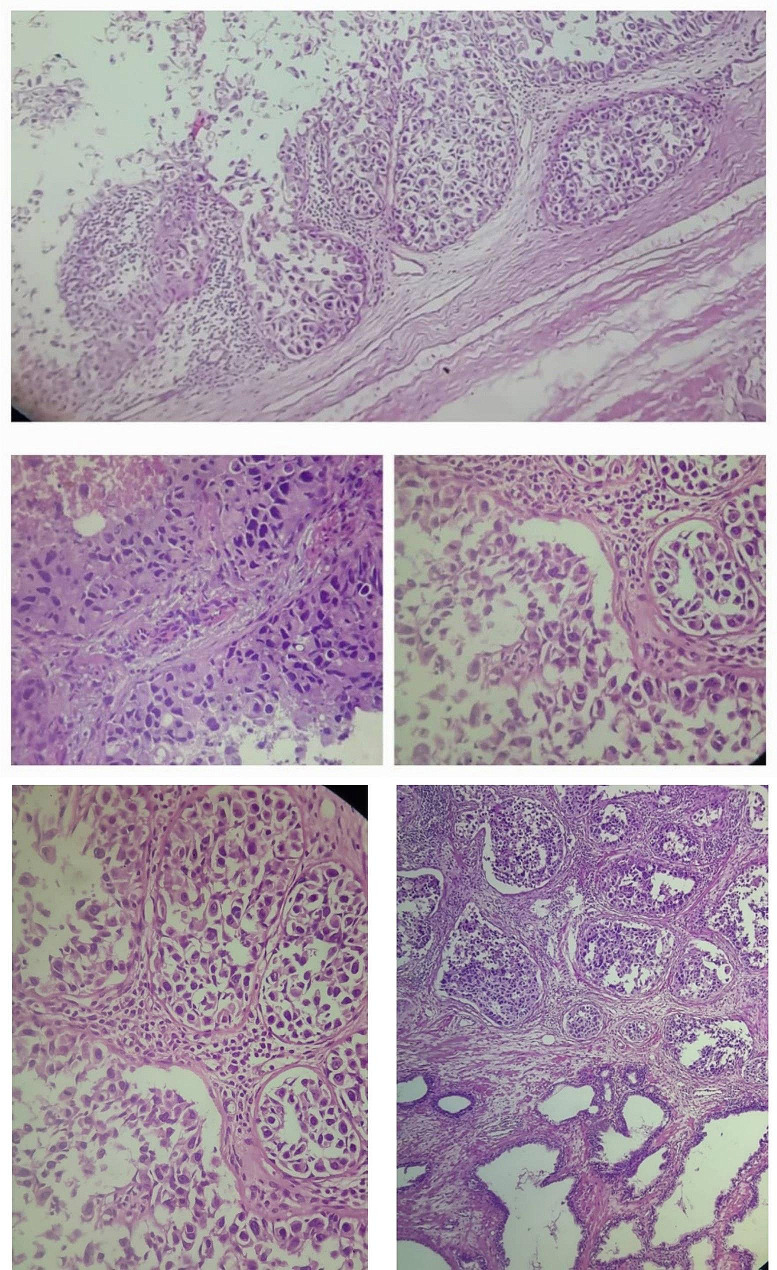
Microscopic examination of prostate glandular tissue showed neoplastic cells (high grade nuclear features: nuclear pleomorphism, hyperchromatic, high N/C ratio with frequent mitotic figures) arranged in irregular nests and invading the prostatic stroma and glands

A 58-year-old non-smoker male was admitted to the urology department following an episode of gross hematuria and persistent microscopic hematuria, without any other lower urinary tract symptoms (LUTS), flank pain, or relevant medical or surgical history; however, his 81-year-old father recently passed away from bladder cancer three months prior. Urinalysis revealed the presence of hemoglobinuria along with leukocytes at 80–90/field and Erythrocytes at 60–70/field. The patient was put on an empirical treatment while awaiting the urine culture results, which ultimately showed no signs of microbial growth. Four weeks after the initial test, a follow-up urinalysis revealed that the inflammation had resolved, yet the presence of microscopic hematuria persisted. Digital rectal examination revealed enlargement of the right lobe of the prostate. Also, the Prostate-Specific Antigen (PSA) level was found to be elevated at 11 ng/ml, surpassing the standard upper limit of 4.0 ng/ml, whereas the free PSA concentration was within normal parameters at 0.95 ng/ml. Cystoscopy revealed edematous changes in the bladder mucosa, characterized by multiple red areas and minor bleeding from the bladder and prostatic, without evidence of malignancy based on the available visual assessment at the time. However, biopsies could not be taken at the time due to the equipment malfunctioning. Subsequently, the patient underwent a transrectal ultrasound-guided biopsy of the prostate, which revealed a high-grade carcinoma within the prostatic ducts. Additional testing with immunohistochemical staining resulted in negative NKX3.1 and positive Gata3 markers, indicative of a primary urothelial origin of the cancer (Fig. 1). In response to these results, another cystoscopy was carried out, uncovering a suspicious lesion in the trigone area of the bladder, which led to a decision to excise the lesion (Fig. 2). During this procedure, more tissue samples were obtained from both the adjacent bladder walls and the prostatic urethra. The histopathological analysis confirmed that the lesion located within the bladder’s trigone was a high-grade, in situ urothelial carcinoma. On the other hand, the biopsies from the prostatic urethra showed benign prostatic tissue without any signs of malignancy. In order to decrease the likelihood of human error, another transrectal biopsy of the prostate’s peripheral zone was acquired, revealing an isolated metastasis to the prostatic ducts. Further evaluation, including a CT scan, 99mTc-MDP bone scan, PET scan, and MRI revealed no other metastasis (Fig. 3). The tumor was classified as T4aN0M0 due to its invasion of a nearby organ, which is the prostate. In consideration of the patient’s pathologic data and overall clinical condition, and a complete surgical removal of the bladder and prostate (radical cystoprostatectomy) was performed using an open surgical technique, accompanied by the creation of an Ileal Conduit for urinary diversion. The histopathological examination showed that the tumor measured 3 cm in greatest dimension while invading the muscularis propria of the bladder as well as the prostatic glands. However, no evidence of malignancy was found within the prostatic urethra, the seminal vesicles, or the lymph nodes in both the iliac and obturator regions (Fig. 4). Three weeks postoperatively, the patient was discharged without complications. He then commenced adjuvant chemotherapy, consisting of Gemcitabine 1000 mg/m2 on days 1 (D1) and D8 and Cisplatin 75 mg/m2 on D1, across four 21-day cycles, with the inclusion of dexamethasone to mitigate side effects. Following the completion of treatment, a CT scan conducted to assess for metastases revealed no such findings, confirming the patient’s good health.

## Discussion

Carcinoma in situ (CIS) of the bladder, first described by Melicow MM. in 1952, is recognized as a malignant bladder condition carrying a significant risk for disease progression and increased mortality due to bladder cancer [4]. It can be identified at various times, including (1) Primary CIS, when there’s no previous bladder cancer history; (2) Concomitant CIS, when it’s found at the same time as non-muscle-invasive (NMIBC) or muscle-invasive (MIBC) bladder cancers; and (3) Secondary CIS, when it’s detected in follow-up in patients who have had bladder cancer before [1]. Having a first-degree relative who has been diagnosed with bladder cancer significantly increases the risk of developing the disease [5]. The patient’s father’s death from bladder cancer further emphasizes the potential hereditary risk factors associated with the condition. While it’s known that smoking can elevate the risk of developing bladder cancer by up to fivefold [1, 3], in this instance, the patient has no history of smoking. Due to the lack of a specific screening test for bladder cancer, the diagnostic process frequently depends upon the recognition of some clinical manifestation in primary healthcare settings [6]. Shephard EA and colleagues’ study among those patients revealed seven distinct characteristics that were independently predictive of bladder cancer, which were gross hematuria, dysuria, urinary tract infection, leukocytosis, abdominal pain, elevated inflammatory markers, and elevated creatinine levels [7]. The Expert Panel of the American Urological Association has described asymptomatic microscopic hematuria as identifying “three or more red blood cells per high-power field” when examining urinary sediment under a microscope, from at least two of three urine samples collected correctly [8]. In this case, the initial urinalysis revealed 60–80 RBCs per field with positive hemoglobin, in conjunction with gross hematuria. A follow-up urinalysis four weeks later still showed 8–10 RBCs per field. And thus, further testing was pursued to determine the cause of the patient’s persistent microscopic hematuria. Roughly 75% of bladder cancer patients exhibit intermittent hematuria that is painless. Studies suggest that around 20% of individuals assessed for gross hematuria will end up with a diagnosis of bladder cancer. In cases where patients have non-visible, microscopic blood traces in the urine, there’s about a 10% chance they will be found to have bladder cancer [3]. The clinical assessment of the spread of UC can be challenging, but combining the histopathological reports with imaging methods such as Ultrasonography (USG), computed tomography (CT), and magnetic resonance imaging (MRI) can aid in determining the size and extent of the primary tumor and identifying any metastases to lymph nodes or distant organs [9]. The occurrence of primary initial CIS of the prostate is exceptionally uncommon. Around 90% of prostatic CIS cases are detected alongside either papillary or invasive urothelial carcinoma (UC), commonly situated in the bladder, as the presence of bladder CIS elevates the risk of concurrent prostatic CIS [2]. Accurately assessing the full extent of CIS within the prostate is crucial. For optimal results, a biopsy should be taken from the prostatic urethra using a resectoscope with a loop electrode, focusing on regions at the 5 and 7 o’clock marks stretching from the bladder neck to the verumontanum, which includes the areas of the ejaculatory ducts [2]. Such a procedure is key to detecting whether the CIS has invaded the prostatic ducts (pdCIS) or infiltrated the prostatic stroma (psCIS), while it’s also noted that in 20% of cases, CIS remains localized to just the prostatic urethra (puCIS) [2]. Stromal invasion is associated with poorer survival compared to non-invasive disease, with a 15-year survival rate of 82% for patients without invasion, whereas it drops to 48% for those with stromal invasion [10]. In this instance, the prostatic urethra was clear of any malignancy; however, a solitary metastasis was identified within the prostatic ducts. The mechanisms behind this selective metastasis may be linked to the anatomy of the prostatic ducts and their fluid dynamics [11], or possibly due to the expression of specific adhesion molecules or growth factors that facilitate the migration of cancer cells to this location [12]. Primary UC typically presents with positive GATA-3 marker results [13], which aligns with the findings in this particular case. This lesion progressed to invade the peripheral zone of the prostate, at which point it was reclassified from CIS, and thus required the implementation of an aggressive approach to treatment [2]. Research comprising numerous trials and meta-analyses regarding radical cystectomy for treating locally advanced bladder cancer strongly supports its efficacy, with a notable increase in the survival rates without recurrence among these patients. The critical factor in achieving optimal outcomes following radical cystectomy is the proficiency with which the procedure is performed [14]. Early cystoprostatectomy in patients with minimal stromal involvement and organ-confined bladder cancer may lead to improved survival rates. Studies by Herr and Donat, as well as Esrig et al., have shown better survival outcomes in patients with prostatic stromal invasion through the intraurethral route (ranging from 64.6 to 75%) compared to non-organ-confined “extravesical” bladder tumors (ranging from 13.6 to 9%) [15]. Radical cystectomy alone is often insufficient to cure locally advanced bladder cancer (T3-T4a N0M0), making adjuvant chemotherapy highly recommended to reduce the risk of recurrence [14]. Both methotrexate/vinblastine/doxorubicin/cisplatin (MVAC) and gemcitabine and cisplatin (GC) chemotherapy combinations are utilized, but GC has demonstrated superior safety and improved 5-year survival statistics, thus it is now favored over the MVAC regimen [16].

## Conclusion

The occurrence of bladder cancer metastasizing to the prostate without involving the prostatic urethra is uncommon. Close monitoring and appropriate treatment interventions tailored to the specific characteristics of the tumor will be crucial for managing the condition effectively and preventing recurrence or progression. Further follow-up and surveillance are recommended to ensure early detection of any potential changes or developments in the disease course.

## Data Availability

The materials used during the current study are available from the corresponding author on reasonable request.
